# Laparoscopic Adnexectomy for Ovarian Torsion during Late Pregnancy: Case Report of a Non-Conservative Treatment and Literature Analysis

**DOI:** 10.3389/fsurg.2017.00050

**Published:** 2017-10-11

**Authors:** Jean Bouquet de Joliniere, J. B. Dubuisson, F. Khomsi, A. Fadhlaoui, G. Grant, N. Ben Ali, A. Major, A. Feki

**Affiliations:** ^1^Départment of Gynecology and Obstetrics and Operative Surgery, Cantonal Hospital of Fribourg (HFR), Fribourg, Switzerland

**Keywords:** ovarian torsion, pregnancy, laparoscopy, ultrasound, adnexectomy

## Abstract

Diagnosis of adnexial torsion is difficult during pregancy (1). The time of decision and laparoscopy is that of the risk of necrosis of the adnexa and, therefore, of the ovarian prognosis. The loss of an ovary can compromise the following fertility. Even if concerns related to laparoscopy in pregnant patients include a limited surgical field, with a risk of uterine injury and negative fetal effects of CO_2_ insufflation. Evidence base suggests that minimally invasive surgery can be safe and better than laparotomy for management of adnexal masses during late pregnancy with good postoperative and obstetric outcomes. If for most authors laparoscopy appears to become the best approach for ovarian torsion during pregnancy (2), nonetheless, the ideal surgical laparoscopic approach of adnexa in late pregnancy remains controversial. Since there is no technical gold standard to overcome surgical difficulties which could make laparoscopic procedures as real challenge in patients in second and third trimester (3); at least, in case of radical and non-conservative treatment, the risk for a first trimester of pregnancy is to remove the corpus luteum (1).

## Case Report

We report a 27-year-old female pregnant with bichorial twin pregnancy, 30 weeks of amenorrhea, after ovarian stimulation for polycystic ovaries, without previous ovarian cyst known. At her admission, she had an acute abdomen, without signs of hyperstimulation but the diagnosis was not immediately posed.

At the entrance, an ultrasonography was performed and a mass of 8 cm × 5 cm was seen in the right adnexal region, without vegetations inside, with doppler abnormalities but with a normal pregnancy.

The contralateral ovary was normal without peritoneal hemorrhage.

The patient did not have fever, had no vaginal bleeding, was without fetal electrocardiogram disorder and a normal contractility, a defense of the right iliac fossa, but with an evident surgical indication of laparoscopy. The differential diagnosis with appendicitis is made but the white blood cells are normal. No tocolysis was prescribed.

We performed an explorative laparoscopy by Veress needle insertion just below the sternum. A 20 mmHg CO_2_ pneumoperitoneum was induced and the laparoscopic view revealed a twisted, edematous right adnexa with an enlarged and necrotic ovary. After inserting two operative trocars on the right side, a first one in the superior and a second one in the inferior quadrant: unfortunetely a non-conservative treatment was performed.

The right adnexectomy was performed. The adnexa was removed through the operative ancillary trocar by an endobag. The postoperative follow-up was uneventful and a normal fetal outcome was confirmed by ultrasound and obstetrical assessment. Histology showed hemorrhagic necrosis with follicles and corpus luteum.

## Literature Analysis

Adnexal torsion is a gynecologic emergency, leading to ischemia and sometimes to ovarian necrosis (4, 5). Its diagnosis can be difficult especially during pregnancy (1). Rather rare, its overall incidence in the literature is 1/10 per 10,000 spontaneous pregnancies (6). Rackow and Patrizio (5) showed that adnexal torsion may occur in 12–25% of pregnant women. It is more common in IVF pregnancies (high size of ovaries because stimulated). Oellsner and Shashar describe adnexial torsion as the fifth most common gynecological emergency (7).

It may involve also fallopian tube or both, due to a partial or complete twisting of the vascular pedicle (4). The obstruction of vein, arteria, and lymphatic channels results in ovarian necrosis.

Due to anatomic reasons, it is described in the literature (8) that the right adnexa is most involved because hypermobility of the right utero-ovarian ligament which is longer than the left.

The ovarian size and weight increase the risk of torsion. After conservative hysterectomy, it is better to fix ovaries as a preventive way to avoid the risk of torsion.

50% of ovarian masses were mature cystic teratomas and corpus luteal cysts (3). In case of IVF ovarian hyperstimulation the risk is high (5).

After ovarian stimulation, the risk of adnexial torsion in pregnancy rises to 6% reaching up to 16% in case of hyperstimulation (9, 10).

The symptoms are not specific but commonly: sudden and acute pain localized on the side of the torsion is described, associated with nausea, abdominal defense generally without fever (2). There are no specific blood markers, only sign of inflammation.

The differential diagnosis is classically appendicitis, PID, adnexal or ovarian cyst, ectopic pregnancy, fibroma necrosis, sigmoid diverticulitis. But these diseases have their own clinical sign by elimination.

We insist on the sudden onset of pain “in a serene sky” during pregnancy. Temperature and blood pressure are normal. Biology is not specific.

The ultrasound signs of adnexal torsion are highly variable (1). But in comparison with a normal ovary the appearance is different (11).

US imaging can reveal ovarian cyst with abnormal position, free peritoneal fluid due to infarction and hemorrhage, and edematous and enlarged ovary.

Doppler blood flow may be normal or abnormal (1). “The whirlpool sign” is the main sign in case of adnexal torsion (12): it must show the ovary twisted around the ovarian ligament or infundibulopelvic ligament as a whirpool or a spiral. The color doppler US may show an absence of arterial and venous blood flow and in this case this is predictive of non-viability of the ovary. This sign increase the diagnosis sensitivity.

Authors describe that in partial or early torsion, blood flow could be maintained (4). In case of absence, the ovary prognosis is really compromised.

In our case, the flow to her ovary was not present.

When acute pain disappears, it is a clinical sign of seriousness in relation to possible ovarian necrosis. Ischemia and after ovarian necrosis are caused by a severe ovarian tissue congestion and a decreasing venous return (8, 13, 14).

At least, in case of ovarian torsion suspected during second and third trimesters of pregnancy, magnetic resonance imaging is useful because it is difficult sometimes to visualize ovaries by a routine ultrasound (15).

The moment of laparoscopy must not be differed to preserve ovary: the surgical treatment must be tried conservative in case of an infertile patient, but unfortunately an adnexectomy is often realized. The literature analysis shows that the conservative treatment is correlated with the time of the surgical indication.

This is the reason why emergency physicians must be trained to avoid a delay in the management and thus preserve the ovary for a future pregnancy.

The surgeon should always try to untwist the adnexa with hot serum and wait for her refill (16–19). Even the ovary or adnexa are black or dark purple, a conservative surgical treatment must be considered (1, 18, 19). The clinical appearance of an ovarian torsion does not correlate well with the residual function (1).

Most articles recommended to wait for 30 min for the recoloration of the appendix before taking a surgical decision.

Washing and aspiration of every peritoneal fluid avoid posterior adhesions and postoperative pain.

## Conclusion

Early diagnosis of adnexal torsion during pregnancy is essential to allow conservative management. Even beyond 16th week of amenorrhea, laparoscopy can be an option unless limited surgical experience, preoperative suspicion of severe abdominal adhesions, or ovarian malignancy. To avoid complications, Veress needle and videolaparoscope should be inserted below the sternum or at the Palmer point if adhesions are suspected on the midline. Operative trocars should be inserted both on the pathologic organ side. If necessary, a third trocar can be inserted on the midline, below the umbilicus, to improve exposure by pushing the uterus aside and atraumatically.

## Author Contributions

All the authors participated in the writing of the article.

## Conflict of Interest Statement

The authors declare that the research was conducted in the absence of any commercial or financial relationships and no potential conflict of interest. The patient accept an anonymous publication.

## References

[1] Mathew M Mubarak SA Jesrani SK. Conservative management of twisted ischemic adnexa in early pregnancy. Ann Med Health Sci Res (2015) 5(2):142–4. 10.4103/2141-9248-153630 25861537 PMC4389332

[2] Zacharoula S Setubal A. Acute abdomen in pregnancy due to isolated fallopian tube torsion: the laparoscopic treatment of a rare case. World J Clin Cases (2014) 2(11):724–7. 10.12998/wjcc.v2.i11.724 25405198 PMC4233421

[3] Koo YJ Lee JE Lim KT Shim JU Mok JE Kim TJ. A 10-year experience of laparoscopic surgery for adnexal masses during pregnancy. Int J Gynaecol Obstet (2011) 113(1):36–9. 10.1016/j.ijgo.2010.10.020 21247562

[4] Zucchini S Marra E Diagnosis of emergencies in gynecology and during the first trimester of pregnancy. J Ultrasound (2014) 17(1):41–6. 10.1007/s40477-013-0059-0 24616750 PMC3945190

[5] Rackow BW Patrizio P. Successful pregnancy complicated by early and late adnexal torsion after in vitro fertilization. Fertil Steril (2007) 87:697. 10.1016/j.fertnstert.2006.05.091 17141765

[6] Turgut A Burak Y. Laparoscopic management of adnexal torsion in a twin, in vitro fertilization pregnancy at 23 weeks. Wideochir Inne Tech Maloinwazyjne (2014) 9(4):655–7. 10.5114/wiitm.2014.45732 25562010 PMC4280421

[7] Oellsner G Shashar D Adnexial torsion. Clin Obstet Gynecol (2006) 49:459–63. 10.1097/00003081-200609000-00006 16885653

[8] Boyd CA Riall TS Unexpected gynecologic findings during abdominal surgery. Curr Probl Surg (2012) 49:195–251. 10.1067/j.cpsurg.2011.12.002 22424211 PMC3313456

[9] Bide D Mashiach S Dulitzky M Surgical and pathologic findings of adnexal torsion in pregnant and nonpregnant women. Surg Gynecol Obstet (1991) 173:363–6. 1948585

[10] Mashiach S Bider D Moran O Adnexal torsion of hyperstimulated ovaries in pregnancies after gonadotrophin therapy. Fertil Steril (1990) 53:76–80. 10.1016/S0015-0282(16)53219-1 2295348

[11] Smorgick N Maymon R Mendelovic S Herman A Pansky M. Torsion of normal adnexa in postmenarcheal women: can ultrasound indicate an ischemic process? Ultrasound Obstet Gynecol (2008) 31:338–41. 10.1002/uog.5194 18247323

[12] Vijayaraghavan SB Sonographic whirpool sign in ovarian torsion. J Ultrasound Med (2004) 23(12):1643–9. 10.7863/jum.2004.23.12.1643 15557307

[13] Hoover K Jenkins TR Evaluation and management of adnexal mass in pregnancy. Am J Obstet Gynecol (2011) 205(2):97–102. 10.1016/j.ajog.2011.01.050 21571247

[14] Hasson J Tsafrir Z Azem F Bar-on S Almog B Mashiach R Comparison of adnexal torsion between pregnant and nonpregnant women. Am J Obstet Gynecol (2010) 202(6):536.e1–6. 10.1016/j.ajog.2009.11.028 20060090

[15] Eskandar O Eckford S Watinson T Safety of diagnostic imaging in pregnancy. Part 2: magnetic resonance imaging, ultrasound scanning and Doppler assessment. Obstet Gynecol (2010) 12:171–7. 10.1576/toag.12.3.171.27599

[16] Damigos E Johns J Ross J An update on the diagnostics and management of ovarian torsion. Obstet Gynecol (2012) 14:229–36. 10.1111/j.1744-4667.2012.00131.x

[17] Descargues G Tinlot-mauger F Gravier A Lemoine JP Marpeau L Adnexal torsion: a report on forty-five cases. Eur J Obstet Gynecol Reprod Biol (2001) 98:91–6. 10.1016/S0301-2115(00)00555-8 11516806

[18] Oelsner G Cohen SB Soriano D Admon D Mashiach S Carp H. Minimal surgery for the twisted ischaemic adnexa can preserve ovarian function. Hum Reprod (2003) 18:2599–602. 10.1093/humrep/deg498 14645177

[19] Mathew M Untwisting and fixation of ovarian torsion in early pregnancy. JABHS (2008) 9:65–7.

